# A Mental Health Management and Cognitive Behavior Analysis Model of College Students Using Multi-View Clustering Analysis Algorithm

**DOI:** 10.1155/2022/2813473

**Published:** 2022-09-27

**Authors:** Danhui Dong, Xiaoying Shen

**Affiliations:** Wuxi Vocational College of Science and Technology, No. 8 Xinxi Road, Wuxi, Jiangsu 214000, China

## Abstract

In this new era that is full of social changes, ongoing economic transformation, an abundance of information resources, and a fast pace of life, the pressure that people feel to compete with one another is also increasing day by day. Because of the vast differences in people's states of consciousness and worldviews, interpersonal relationships have become increasingly difficult to navigate. Students in higher education institutions will eventually emerge as the dominant demographic in society. Their mental health has a significant bearing on all aspects of life, including learning and future growth. An objective condition that must be met in order to guarantee that the next generation of talent will have a high level of overall quality is the improvement of the mental health of college students (CSMH) in the new era. One component of public health is the emotional well-being of students in higher education. The state of the public's health is consistently ranked among the most urgent problems facing modern society. However, there is not much hope for the Chinese CSMH. In order to effectively manage their mental health, a variety of educational institutions, including colleges and universities, have proposed a large number of management strategies for CSMH. The vast majority of these strategies are not targeted, and they do not offer a variety of management strategies that are based on the many different psychological states. It is necessary to first be able to accurately predict the mental health status of each individual college student in order to achieve the goal of improving the mental health management of students attending colleges and universities. This study proposes using a multi-view K-means algorithm, abbreviated as MvK-means, to analyze the CSMH's data on mental health. This is possible because the data can be obtained from multiple perspectives. This paper presents a multi-view strategy as well as a weight strategy in light of the fact that each point of view contributes in its own unique way. Different weight values should be assigned to each view's data, which will ultimately result in an improved evaluation effect of the model. The findings of the experiments indicate that the model that was proposed has a beneficial impact on the analysis of the data pertaining to the mental health of college students.

## 1. Introduction

Students have a unique opportunity to shine on the college stage. College students have reached a point where their physical development is comparable to that of adults, but their mental health has not reached this level of development yet. The atmosphere that the students have been exposed to up to this point has been one that is comparable to the actual social atmosphere. When college students are forced to balance the demands of school, life, and work, their mentalities will shift in a variety of different ways. A number of psychological issues could arise if the various pressures the students are subjected to are not alleviated in a timely and appropriate manner. If these issues are not resolved in a timely manner, it will lead to other psychological issues, which will interfere with the student's ability to study and function in daily life while in college as well as in the future. Students at a wide variety of colleges and universities have been involved in a disturbingly high number of cases of self-harm and suicide in recent years. Some students are unhappy as a result of a disagreement with a fellow student who resides in the same dorm or attends the same class, and finally, as a consequence of a few insignificant occurrences, there is a wounding incident. Some students are subjected to a variety of pressures, and because they are unable to concentrate on it for an extended period of time, they are at risk for committing suicide. All of these predicaments can be traced back to the mental health issues that are prevalent among college students. The psychological well-being of students in higher education falls under the umbrella of the field of public health. Because both the nation as a whole and each individual family must invest a significant amount of time and resources to educate a college student, it would be a terrible waste if something were to happen to him or her before he or she could integrate into society and appreciate the significance of his or her own life. The problems with CSMH have now become a public health concern. As a result of this, it is clear that educational institutions, such as colleges and universities, ought to pay attention to CSMH [1–3] and efficiently manage the data pertaining to the mental health of college students. Problems with mental health have a negative impact not only on the sound physical and mental development of individuals but also on the growth of the nation and society as a whole. As a consequence of this, one of the most important responsibilities of modern college education is to pay attention to and investigate the psychological state of college students, to promptly diagnose and treat any mental illnesses that may be present in college students, and to provide appropriate direction to college students so that they can overcome psychological barriers and treat mental illnesses.

The research that is carried out on the mental health management of college students has the potential to enrich and improve the system that manages mental health. Countries from Europe and the Americas quickly established themselves as pioneers in the field of school mental health management as early as the eighteenth century. It is possible to attribute the rapid progress that has been made in the management of students' mental health in developed countries like the United States and Europe to the fact that the government in these countries is the primary driving force behind these advancements. On the other hand, both the connotation and the expansion of mental health management are extremely complex, and the research results frequently lack unity and the ability to be applied because they are based on hazy concepts. This is because the concepts on which they are based are not well understood. Due to the fact that the starting point for this research was so low, the research on the mental health management of Chinese college students got off to a very sluggish start in China. As a result, the country's research on the topic is far behind schedule. The content of the research has many instances of repetition, and the ideas and methods that were utilized in the research are not in a position to adequately reflect the robust scientific nature of the research. This, in turn, causes inconsistencies in the findings of the research. The majority of the research that is being done on the mental health management system of college students is research that is being done on the mental health services that are being provided to college students. This is because the mental health management system of college students is the focus of the majority of the research that is being done. On the other hand, there is no research being done on the administration of college students' mental health from the point of view of public health. The scope of studies that are relevant is not overly broad, and the majority of the investigations are limited to relatively small samples. These studies have not demonstrated a fundamental understanding of the development law and characteristics of CSMH, nor have they suggested any effective public health management measures to improve CSMH. In addition, these studies have not suggested any concrete actions that could be taken to improve CSMH. Researchers are required to comprehend the structure of mental health management from a variety of perspectives in order to support it with varying degrees of theoretical results. This is necessary because the structure of mental health management is so open. Investigation into this topic has the potential to contribute significantly to the theoretical underpinnings of open systems approaches to the management of mental health. Research in this area is very important due to the fact that the management of the mental health of college students is an important part of the system that manages public health. The research that is being done on the mental health management system of college students is helpful in the development of the mental health management system on a more local level. The idea of a management system for mental health can be segmented into a variety of different levels and organizational structures. Due to the wide variety of cultural settings in which various strategies for managing mental health first emerged, these strategies can take on a wide variety of forms across different cultures. As a direct consequence of this, the research topic of mental health management incorporates an important element of ethnic flavor. It is especially true for a country that is composed of more than one ethnic group with each ethnic group having its own culture, and this is true even more so for the country as a whole. In addition, research on the mental health management system in China is still in its infant stages, and the majority of the studies that have been carried out thus far are of a substandard quality. At this time, the majority of them continue to merely copy foreign theory and practical experience without making any attempts at innovation or transformation in order to produce a distinctive theoretical system that is able to accommodate their very own growth. This is the case despite the fact that there are numerous opportunities for them to develop their own unique perspectives and perspectives that are grounded in their own experiences. In light of this, research into community-based mental health care management in China can be of assistance in working toward the goal of establishing mental health management at the local level.

The majority of CSMH's data collection is done through the use of questionnaires and the collection of physiological data. The traditional approach to managing mental health focuses primarily on the fundamental processes of adding new data, removing old data, and making modifications to existing data. A comprehensive data mining and analysis is not carried out using this method. As a result, it does not have much of an impact on the way CSMH is managed. It is essential to organize and analyze data regarding the mental health of students in order to realize improvements in the efficacy of mental health education provided in schools. The psychological data of college students can be mined in-depth using a variety of methods, such as classification algorithms [4–6], regression algorithms [7–9], the cluster analysis method [10–12], big data technology [13–15], and so on. These techniques are able to perform an accurate analysis of the issues concerning the psychological information of the students. The potential and implicit information in the data can be obtained by relying on the platform of the psychological management system, which is combined with data mining technology. This allows for a large amount of data to be analyzed, which in turn helps obtain potential and implicit information. This kind of implicit information will provide major universities with a corresponding reference basis and effective solutions. Wearable technology and social networking software are utilized in the data collection process for reference [16]. The information is then entered into a system that makes predictions about people's mental health, which allows for the prediction of the mental health of each individual student. The purpose of the research cited in reference [17] is to identify people who have psychological issues by conducting an analysis of social data using natural language processing methods and machine learning algorithms. Algorithms that are used in machine learning are utilized in reference [18] to identify mental health states and to make predictions regarding illness. Over 50 previous studies on the application of machine learning in mental health assessment were analyzed for this study. The author provides a summary of the current state of research on mental health that is based in machine learning techniques, using the studies mentioned above as support. Deep learning algorithms are used in reference [19] to mine the social data of individuals in order to determine the individuals' mental health status. The mental health state evaluation method that is based on the deep learning algorithm has the potential to have an accuracy of evaluation that is close to 90%. In reference [20], typical machine learning algorithms are utilized in the assessment of a person's mental health status, and the results of the assessments obtained by a variety of conventional machine learning algorithms are compared to one another.

The primary focus of the research work that was described above is the examination of data pertaining to health that was obtained from various social networking platforms. These data include some information that is either inaccurate or unqualified, both of which will have an effect on the final results of the mental health assessment. In addition, the majority of the studies referred to above conduct evaluations of psychological states by making use of professional evaluation tools, machine learning algorithms, and deep learning algorithms. There is room for advancement in terms of the accuracy of the evaluation that was obtained as a result of the method that was utilized. In light of the arguments that were presented earlier, the purpose of this paper was to propose a model for the management of the mental health of college students that is based on an algorithm for multi-view clustering. Specifically, the paper will discuss how this model will work. The following is an outline of the most important contributions made by this study: (1) When analyzing students' mental health, we use both the data on the students' basic information as well as the data from the questionnaires. (2) An algorithm called MvK-means has been proposed with the intention of being used for the purpose of training a mental health management model. The default behavior of the traditional multi-view algorithm is to give equal weight to each view. This is one of the available settings. When dealing with real data, it is not appropriate to use this strategy, which presumes that each view has the same quality by default. This is due to the fact that the quality of the real data varying greatly from case to case. In light of this, a weighting strategy has been implemented in this piece of writing in order to effectively control the significance of each viewpoint. (3) Feed the collected data into the trained model in order to obtain information regarding the mental health status of college students who are enrolled in the educational institution. As a direct result of the information contained in this report, CSMH is being managed in an efficient manner. Experiments have shown that the model for the management of college students' mental health that is based on the multi-view clustering algorithm that is used in this paper is capable of producing better results. This paper utilizes the model.

## 2. College Students' Mental Health Management from the Standpoint of Public Health

### 2.1. College Students' Mental Health Standards and Management

The idea of mental health standards is presenting a dynamic development trend as a direct result of the progress that has been made in human society, the economy, and science. This is the case because the concept of mental health standards is presenting a dynamic development trend. The ten mental health standards that were proposed jointly by Maslow and Mittelman are among the standards that have the greatest amount of public recognition. The standards provide a comprehensive breakdown of the various aspects of security, such as an accurate assessment of one's own capabilities, the development of an ideal personality, the mastery of one's feelings, the enhancement of one's connections with others, and the promotion of personal development. “Physical, intellectual, and emotional mediation, adapting to one's environment, having a sense of well-being, giving full play to one's abilities at work, and living a productive life,” was how the International Conference on Mental Hygiene defined mental health in 1946. At the time, this was considered to be the standard for mental health. Over the past few years, academics in China have increased the amount of research they have done on the CSMH and have proposed new standards for the CSMH. College students should have the following mental health standards, according to the general consensus among all students: correct three views; personality integrity; positive mood; positive and harmonious interpersonal relationships; objective self-evaluation; positive sense of competition; love of life; willingness to learn; and the courage to pursue life value. Defining the mental health standards that college students should achieve is the first and most important step toward improving the level of mental health management among college students.

Management is the process of effectively planning, organizing, leading, and controlling the resources owned by an organization within a specific environment in order for the organization to achieve the goals that have been set for the organization. These goals have been set in order for the organization to be successful. The term “CSMH management” refers to the related plans that have been formulated by higher education management institutions and schools to improve the mental health status of college students and improve the mental health level of college students according to the current situation of CSMH. The goal of these plans is to both improve the mental health status of college students and improve the mental health level of college students. The mental health of college students is going to be prioritized in the implementation of these plans. We can establish a special management department to provide psychotherapy and management for these college students who have mental health issues. The content, personnel, mechanism, mode, and measures of mental health management are the primary focal points of investigation in the studies that are currently being conducted on the topic of the management of the mental health of Chinese college students. These studies are being carried out in order to shed light on the topic of the management of the mental health of Chinese college students. There are not many studies that concentrate on the particular objectives or staged objectives of management, regulatory means, or specific evaluations. Therefore, the establishment of CSMH organization and management mechanism and the improvement of the relevant management system are issues that need to be accelerated in the academic circles. Also, these are questions that need to move much more quickly through the academic community. This study was conceived with the intention of both improving the mental health management system and bringing attention to the significant role that management plays within the mental health management system. The aforementioned circumstances served as the impetus for the development of this study.

### 2.2. The Principles of Mental Health Management of College Students

**Figure 1:**
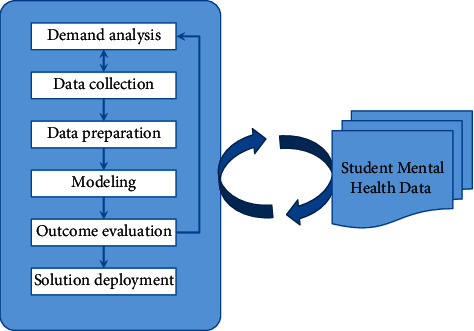
The CSMH management principle diagram.

An in-depth analysis and application of CSMH data serve as the bedrock of efficient CSMH management. After data on the mental health of college students have been collected through a variety of channels, an intelligent model is used to analyze the data and extract potentially informative information from the data in order to evaluate the psychological state of the students. The findings of the evaluation are incorporated into the development of intervention strategies for the treatment of mental health issues experienced by college students. This strategy has the potential to improve the effectiveness and efficiency with which universities manage their student bodies. Specifically, it has the potential to improve the effectiveness of universities. The following is an illustration of the principle for the management of the mental health of college students, which can be found in Figure 1:

The CSMH management process is depicted as being a component of the data mining workflow in the figure. The entirety of the procedure is connected by five links, which are as follows: demand analysis, data collection, data sorting, modeling, and the results of data evaluation.

1. The purpose of the stage of demand analysis is primarily to determine what useful information will be unearthed from the mental health data of college students during the course of this research. A well-defined objective serves as the foundation of demand analysis. The primary goal of this study was to gain a comprehensive understanding of college students' mental health status by analyzing data on college students' mental health. Students who may have psychological issues receive early warning, and professional psychological counselors will timely and effectively guide them according to the warning information they receive. After the objective has been defined, the necessity of this research can be summed up as the identification of psychological issues, either imminent or ongoing, that are present in college students. Once a diagnosis has been established, the appropriate medication can be prescribed based on the most successful approaches to the management of mental health.
2. The information data are the raw material, and because the raw material is of such high quality, the final product will also be of such high quality. As a result, the data collection process ought to be as exhaustive, in-depth, and precise as is humanly possible. For the purpose of collecting data on CSMH, it is possible to start with the fundamental information of the students, social data, psychological questionnaires, and so on. Collect data related to CSMH from a variety of perspectives in order to enrich the information collected.
3. Data sorting. In addition to the collection of raw data, preprocessing of that data is necessary. When it comes to the raw data that were collected, some of them are invalid data, some of them are incomplete data, and some of them are redundant data. The presence of these data will have a significant impact on the data mining process's outcome. As a result, the importance of organizing the data cannot be overstated.
4. Create an evaluation model. Models for data mining can be trained and created based on a selection of algorithms. A multi-view clustering algorithm was utilized for the purposes of this study. The execution steps of the algorithm consist of training a mental health assessment model using the training dataset. This model is then used to evaluate patients.
5. Result evaluation. The trained model is then given the test dataset to analyze, which allows the performance of the trained model to be evaluated. Calculated based on the evaluation index is the model's overall performance rating in terms of its evaluation. If the evaluation results are very positive, this indicates that the trained model is performing very well. The evaluation results that are produced by the model can provide college administrators with assistance in effectively managing college students.

### 2.3. Architecture of CSMH Management System

**Figure 2:**
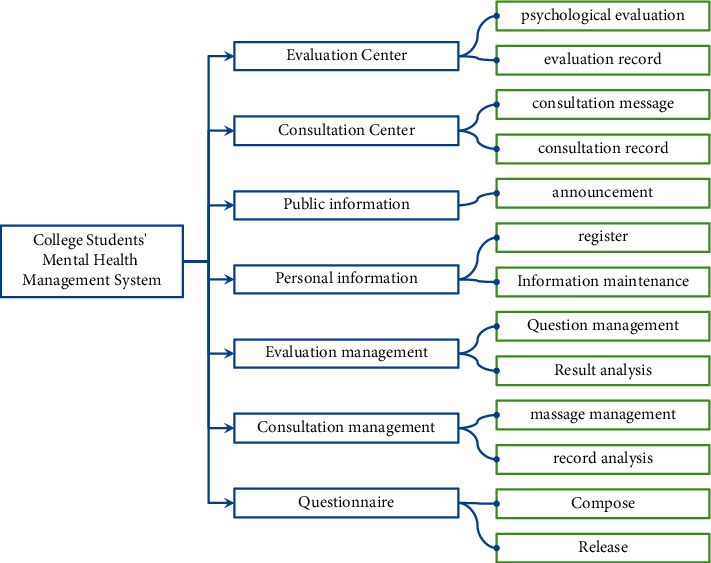
Architecture of CSMH management system.

A complete mental health management system for college students should have psychological evaluation center, evaluation management, counseling center, counseling management, public information, personal information, and other functions. Among them, the evaluation center has the functions of collecting test evaluation data, psychological evaluation, and evaluation record management. Evaluation management includes evaluation data management, combined evaluation management, and evaluation result management. The consultation center includes consultation message, online consultation, and appointment consultation. Consulting management includes appointment management, appointment scheduling, and message management. Public information includes viewing announcement information, mental health guide, public information management, and psychological article management. Personal information mainly includes registration, login, password retrieval, and personal information maintenance. Other functions mainly include file management and information management.  Figure 2 depicts the system's architecture.

### 2.4. Classical K-Means Clustering Algorithm

**Figure 3:**
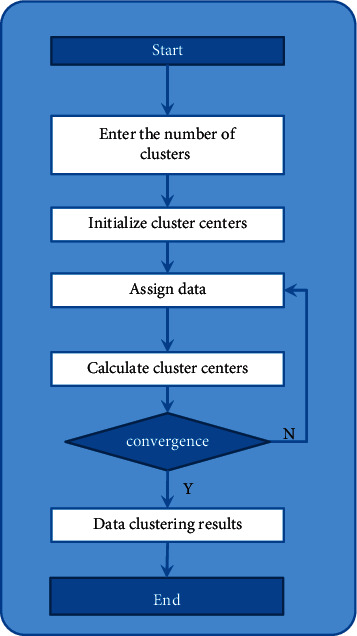
K-means algorithm flow chart.

On all of the data, the K-means algorithm is used to perform distance-based clustering. The primary objective of this algorithm is to determine the cluster center points for each classification with as much specificity as is feasible. Assume there are *k* different categories and *n* total data points. Calculate the distance between each sample and each class center, then choose *k* data at random to serve as the initial cluster center, calculate the distance between each sample and each class center, and divide the remaining data into classes as close to the class center as possible. It is necessary to continuously calculate the distance between each sample and a new round of cluster centers throughout the entire process, which is an iterative update process. The more iterations that are performed, the better the cluster centers that are obtained, up until the point where the cluster centers are no longer variable, consequently gaining possession of the last available class center. Following the selection of the class center, the members of each class are chosen.  Figure 3 depicts the detailed steps of the K-means clustering model [21], which are as follows:

The objective function of the K-means algorithm is as follows. The Euclidean distance is used in the function to calculate the distance between each data point and the cluster center.

(1) $$\begin{array}{c} J=\overset{k}{\underset{i=1}{\sum }}\overset{n}{\underset{j=1}{\sum }}w_{ij}d_{ij}^{2}, \end{array}$$

where *k* is the number of categories in the dataset and *n* is the total number of samples in the dataset. *d*_*ij*_=‖*x*_*i*_ − *z*_*j*_‖ is used to calculate the Euclidean distance. *x*_*i*_ represents the *i*th sample and *z*_*j*_ represents the *j*th class center. *w*_ij_ represents the class to which the data point belongs.

(2) $$\begin{array}{c} w_{ij}=\left\{\begin{array}{c} 1,\;\\ 0.\; \end{array}\right. \end{array}$$

If *w*_*ij*_is 1, it means that the *j*th data point belongs to the class *S*_*i*_. When *w*_*ij*_ is 0, it means that the *j*th data point does not belong to the *S*_*i*_ class.

Equation (1) translates to the following expression:

(3) $$\begin{array}{c} J=\overset{k}{\underset{j=1}{\sum }}\underset{x_{i}\in S_{i}}{\sum }d_{ij}^{2}, \end{array}$$

where *d*2/*ij* represents the sum of the squared errors of the data points and the corresponding cluster centers. Therefore, the objective function is also called the error squared sum criterion function.

In order to obtain the cluster center *cj* of each update, the following update iterative formula can be obtained according to the optimization theory of Lagrangian conditional extrema:

(4) $$\begin{array}{c} z_{j}=\frac{1}{n}\underset{x_{m}\in S_{j}}{\sum }x_{m}\;j=1,2,3,\ldots ,k. \end{array}$$

## 3. Mental Health Assessment Models

### 3.1. Multi-View Clustering Model

**Figure 4:**
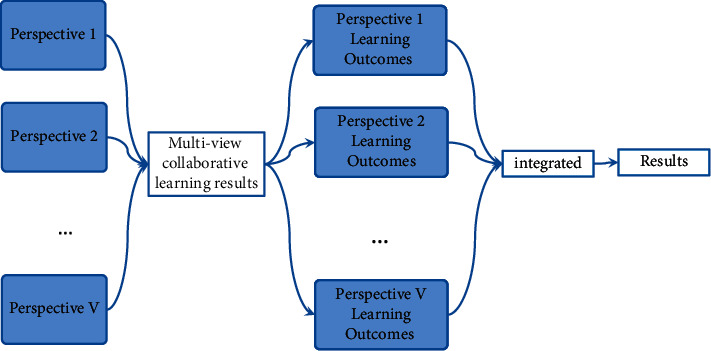
Multi-view learning model.

A more effective multi-view clustering technology has been discovered through the investigation of traditional clustering analysis methods. The technology allows multi-view data with multiple features to learn collaboratively in the clustering process, solving the problem of complex data with multiple features. Traditional clustering algorithms may be limited to dealing with a single feature of complex data. Early multi-view clustering techniques took into account each view of the data and treated each view as a separate clustering task. After obtaining the clustering results for each view, the ensemble learning mechanism is used to select an appropriate ensemble learning strategy, and the results of multiple views are combined to produce the final marriage result. It has been discovered that multi-view learning makes use of the connections between various features in the dataset by studying various models of multi-view algorithms [22]. Make full use of the differences and correlations between various points of view so that the final learning outcomes are consistent.  Figure 4 depicts the multi-view learning model.

### 3.2. Multi-View K-Means Algorithm

The evaluation center is the central component of the mental health management model for college students. The evaluation center is primarily used to collect CSMH data and enter it into the trained mental state evaluation model. The model uses the evaluation results to determine each college student's mental state. The most important of these is evaluating the model. CSMH data include many aspects, such as social data, questionnaires, academic data, personal basic information, and so on. As a result, this paper intends to train the evaluation model using a multi-view learning model.

In order to represent the data with multiple features by K-means, a weight vector *W* is assigned to the clustering of each view, and *W* satisfies the following conditions:

(5) $$\begin{array}{c} \overset{V}{\underset{v=1}{\sum }}w_{v}=1,0\leq w_{v}\leq 1,0\leq v\leq V. \end{array}$$

A multi-view dataset *X* has *N* samples and *V* views, *X*={*x*_*i*_}_*i*=1_^*N*^. *x*_*i*_={*x*_*i*_^(*v*)^}_*v*=1_^*V*^, *x*_*i*_ ∈ *R*^*d*(*v*)^ is the view vector of sample *x*_*i*_. The objective function of MvK-means is as follows:

(6) $$\begin{array}{c} {\overset{V}{\underset{v=1}{\sum }}\overset{C}{\underset{j=1}{\sum }}\overset{N}{\underset{i=1}{\sum }}w_{v}^{p}\delta_{ij}\left(x_{i}^{\left(v\right)}-z_{j}^{\left(v\right)}\right)}^{2}. \end{array}$$

*z*_*j*_^(*v*)^=(*z*_*j*1_^(*v*)^, *z*_*j*2_^(*v*)^, ..., *z*_*jN*_^(*v*)^) is the *i*th cluster center. The number of clusters is *C*, and the total number of data is *N*. *w*_*v*_^*p*^ is the weight vector of the *v*th view, and *p* is the weight index.

Since the quality of each view is different, the algorithm needs to assign a corresponding weight value to each view to represent the importance of the view. When the data of a certain angle of view is scattered, or when a certain angle of view is greatly interfered by noise, it means that the quality of the angle of view is low, and the weight of the data of this angle of view is assigned 0. The scalar *η* is introduced in this paper; when *η* belongs to the *k*th cluster, it is equal to 1, otherwise it is equal to 0. The MvK-means algorithm's objective function is as follows:

(7) $$\begin{array}{c} J_{H}={\overset{V}{\underset{v=1}{\sum }}\overset{C}{\underset{i=1}{\sum }}\overset{N}{\underset{j=1}{\sum }}w_{v}^{p}\phi_{ij}\left(x_{j}^{\left(v\right)}-z_{i}^{\left(v\right)}\right)}^{2}+\eta \overset{V}{\underset{v=1}{\sum }}w_{v}^{p}. \end{array}$$

Using the Lagrange multiplier optimization method to minimize equation (5), the cluster center *z*_*j*_ of the MvK-means algorithm is obtained, and the iterative expression of the weight vector *w*_*v*_ is: Algorithm 1

(8) $$\begin{array}{c} z_{j}^{\left(v\right)}=\frac{\overset{N}{\underset{i=1}{\sum }}\phi_{ij}x_{i}^{\left(v\right)}}{\overset{N}{\underset{i=1}{\sum }}\phi_{ij}}, \end{array}$$

(9) $$\begin{array}{c} w_{v}={\frac{1}{\overset{V}{\underset{v=1}{\sum }}\left[{\sum_{i=1}^{N}\phi_{ij}\left(x_{i}^{\left(v\right)}-z_{j}^{\left(v\right)}\right)}^{2}+\eta /{\sum_{i=1}^{N}\phi_{ij}\left(x_{i}^{\left(v\right)}-z_{j}^{\left(v\right)}\right)}^{2}+\eta \right]}}^{1/\left(p-1\right)} \end{array}$$

## 4. Experimental Results

### 4.1. Experimental Data Collection

In order to analyze the performance of the psychometric model used in this paper, the model needs to be trained. Therefore, this study collected the mental health data of a total of 800 students in computer-related majors from freshman to senior year in a university. Mental health data mainly include basic information of students and questionnaire data. The basic information is shown in Table 1. The comparison table for converting each information in Table 1 into numerical value is shown in Table 2. The questionnaire design is shown in Table 3. The two kinds of information shown in Table 1 and Table 3 are taken as two viewing angle data, respectively. A total of 800 samples. Among them, 600 samples are used as training set and 200 samples are used as the test set.

### 4.2. Experimental Results and Analysis

This section assesses the proposed mental state assessment model's performance on CSMH data. The comparison models used are traditional K-means and fuzzy C-means (FCM). The evaluation indicators use Normalized Mutual Information (NMI) and Rand index (RI). The number of clusters is set to 5, which represent extremely poor, poor, medium, average, and good mental states, respectively. The system generates the initial cluster centers at random. Table 1 shows the attributes of view 1 data, and a total of 8 attributes are used as input variables. Table 3 displays the attributes of view 2 data, and a total of 8 attributes are used as input variables. There is a limit of 100 iterations.

**Figure 5:**
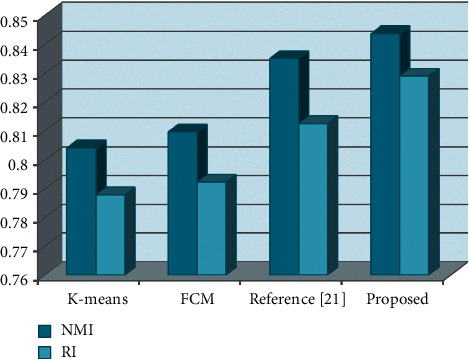
Comparison of experimental results.

In order to calculate the evaluation index, the real psychological state of each student must be known. As the standard data, this paper uses the student SCL-90 symptom self-rating scale to obtain the psychological state results of 800 college students. The experimental results obtained by each model are shown in Table 4 and Figure 5:

The results show that the model proposed in this paper produces optimal evaluation results. Both reference [21] and the model in this paper belong to the multi-view model, and the experimental results show that the experimental results obtained by the multi-view model are significantly better than the single-view learning models such as K-means and FCM. FCM performs slightly better than K-means due to the fact that FCM introduces membership, which reduces the impact of suffering data on the final result. The proposed model performs better than reference [21] because the proposed model introduces a weighting strategy, and each view data will be given corresponding weights according to its own contribution to the results. The weighting strategy can improve the contribution of high-quality views without losing useful information in low-quality view data.

## 5. Conclusion

There are many college students, but fewer college teachers and student managers. In this case, the teacher is unable to accurately understand each student's psychological state. College students' psychological problems are becoming more visible as the environment in which they live becomes more complex and pressure increases. The specific manifestation is a daily increase in the number of college students who have poor mental health. The mental health of college students has a direct impact on the quality of talent training and is linked to the country's future development. As a result, people from all walks of life should pay close attention to how CSMH is managed. Furthermore, relevant mental health management institutions should prioritize CSMH management. Only in this manner will we be able to cultivate high-quality talent capable of adapting to the country's ongoing development in the twenty-first century. As a result, the MvK-means model is proposed in this paper for assessing CSMH. This model is unique in that it employs a multi-view strategy and assigns different weights to different views based on their contribution. The experimental results show that the model has some advantages in assessing college students' mental health. Colleges and universities can implement various management strategies for students with poor mental health based on accurate assessment results. This study's work has the following limitations. First, the data gathered are insufficiently comprehensive. This study primarily collects data from science and engineering students, and the status of CSMH in various major categories will vary. Second, data preprocessing should be improved. To improve data compactness, attributes with high contribution should be selected as much as possible, while attributes with low contribution should be discarded. Third, the model can be improved further to improve the model's evaluation performance. This study will conduct additional research and optimization on the three problems listed above in the future.

## Figures and Tables

**Algorithm 1:**
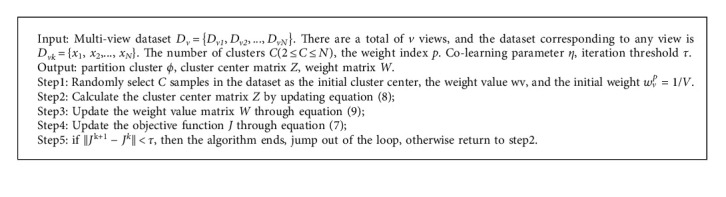
The algorithm flow is as follows.

**Table 1:** Basic information of students.

Gender	Grade	Character	Household income	From rural/urban	Is it an only child?	GPA	Attendance in class
Male	Freshman	Outgoing	Middle	Urban	Yes	Excellent	Good
Female	Junior year	Introverted	Low	Rural	No	Good	Excellent

**Table 2:** Conversion table.

Attributes	Value	Code
Gender	Male	11
	Female	12

Grade	Freshman	21
	Sophomore	22
	Junior year	23
	Senior year	24

Character	Introverted	31
	Outgoing	32
	Inside and outside	33

Household income	Low	41
	Middle	42
	High	43

From rural/urban	Rural	51
	City	52

Is it an only child?	Yes	61
	No	62

GPA	Failed	71
	Pass	72
	Medium	73
	Good	74
	Excellent	75

Attendance in class	Failed	81
	Pass	82
	Medium	83
	Good	84
	Excellent	85

**Table 3:** Questionnaire.

No.	Question	Options
1	Gender	A. male B. female
2	Grade	A. freshman B. sophomore C. junior D. senior
3	Do you feel stressed?	A. very large B. large C. average D. no pressure
4	Source of stress	A. study B. love C. interpersonal communication D. high self-demanding E. do not know F. no pressure
5	How to deal with mental problems	A. talk with friends B. talk with parents C. solve it by yourself D. let it go. E other
6	Whether to attend a mental health education seminar or class?	A. never participated B. listened occasionally C. often participated
7	How to reduce stress?	A. sleeping B. listening to music C. exercising D. talking. E other
8	Can mental health be managed?	A. can B. cannot

**Table 4:** Evaluation results obtained by each model.

Index/model	K-means	FCM	Reference [21]	Proposed
NMI	0.8041	0.8096	0.8351	0.8437
RI	0.7879	0.7923	0.8125	0.8290

## Data Availability

The labeled dataset used to support the findings of this study can be obtained from the corresponding author upon request.

## References

[1] Rosenberg S., Mendoza J., Tabatabaei-Jafari H., Carulla L. S. (2020). International experiences of the active period of COVID-19 - mental health care. *Health Policy and Technology*.

[2] Gibbons A., RoseMahoney M., Joyce Y. C. (2021). The utility of self-perceived health ratings in screening volunteers for mental health research. *Psychiatry Research*.

[3] Zifkin C., Montreuil M., Beauséjour M. È., Picard S., Gendron C. L., Franco A. (2021). Carnevale, an exploration of youth and parents’ experiences of child mental health service access. *Archives of Psychiatric Nursing*.

[4] Ahmed E., Ahmad S., El-Dakhakhni W. (2022). Machine learning classification algorithms for inadequate wastewater treatment risk mitigation. *Process Safety and Environmental Protection*.

[5] Yusof N. M., Muda A. K., Pratama S. F., Carbo-Dorca R., Abraham A. (2022). Improved swarm intelligence algorithms with time-varying modified Sigmoid transfer function for Amphetamine-type stimulants drug classification. *Chemometrics and Intelligent Laboratory Systems*.

[6] Ghazali S. M., Alizadeh M., Mazloum J., Baleghi Y. (2022). Modified binary salp swarm algorithm in EEG signal classification for epilepsy seizure detection. *Biomedical Signal Processing and Control*.

[7] Waqas J., Bouchachia A. (2022). Iterative ridge regression using the aggregating algorithm. *Pattern Recognition Letters*.

[8] Vyas U. B., Shah V. A. (2022). Differential evolution based regression algorithm for mathematical representation of electrical parameters in lithium-ion battery model. *Journal of Energy Storage*.

[9] Chimere O., Ezuma B. S., Yining Lu M. D. (2022). Camp, A machine learning algorithm outperforms traditional multiple regression to predict risk of unplanned overnight stay following outpatient medial patellofemoral ligament reconstruction. *Arthroscopy, Sports Medicine, and Rehabilitation*.

[10] Caruso G., Gattone S. A., Fortuna F., Di Battista T. (2021). Cluster Analysis for mixed data: an application to credit risk evaluation. *Socio-Economic Planning Sciences*.

[11] Francisco D. (2012). Poor mental health symptoms among Romanian employees. A Two-Step Cluster analysis. *Procedia - Social and Behavioral Sciences*.

[12] Marinucci A., Grové C., Allen K.-A., Riebschleger J. (2021). Evaluation of a youth mental health literacy and action program: Protocol for a cluster controlled trial. *Mental Health & Prevention*.

[13] Rubeis G. (2022). iHealth The ethics of artificial intelligence and big data in mental healthcare. *Internet Interventions*.

[14] Mitroshin P., Shitova Y., Shitov Y., Vlasov D., Mitroshin A. (2022). Big data and data mining technologies application at road transport logistics. *Transportation Research Procedia*.

[15] Marcia Maja M., Letaba P. (2022). Towards a data-driven technology roadmap for the bank of the future: exploring big data analytics to support technology roadmapping. *Social Sciences & Humanities Open*.

[16] Liu S., Vahedian F., Hachen D. (2021). Heterogeneous network approach to predict individuals’ mental health. *ACM Transactions on Knowledge Discovery from Data*.

[17] Skaik R., Inkpen D. (2020). Using social media for mental health surveillance: a review. *ACM Computing Surveys*.

[18] Thieme A., Belgrave D., Doherty G. (2020). Machine learning in mental health: a systematic review of the HCI literature to support the development of effective and implementable ml systems. *ACM Transactions on Computer-Human Interaction*.

[19] Kirkpatrick K. (2022). Artificial intelligence and mental health. *Communications of the ACM*.

[20] Thieme A., Belgrave D., Sano A., Doherty G. (2020). Machine learning applications: reflections on mental health assessment and ethics. *Lnteractions*.

[21] Gupta A., Datta S., Das S. (2018). Fast automatic estimation of the number of clusters from the minimum inter-center distance for k-means clustering. *Pattern Recognition Letters*.

[22] Xu R. (2022). The relationship between psychological quality education and mental health level of college students by educational psychology. *Frontiers in Psychology*.

